# Biotinylated Photocleavable Semiconductor Colloidal Quantum Dot Supraparticle Microlaser

**DOI:** 10.1021/acsanm.4c00668

**Published:** 2024-04-16

**Authors:** Charlotte J. Eling, Natalie Bruce, Naresh-Kumar Gunasekar, Pedro Urbano Alves, Paul R. Edwards, Robert W. Martin, Nicolas Laurand

**Affiliations:** 1 Institute of Photonics, Department of Physics, SUPA, University of Strathclyde, Glasgow G1 1RD, U.K.; 2 Fraunhofer Centre for Applied Photonics, 99 George Street, Glasgow G1 1RD, U.K.; 3 Department of Physics, SUPA, University of Strathclyde, Glasgow G4 0NG, U.K.; 4 Institute for Compound Semiconductors, School of Physics and Astronomy, Cardiff University, Cardiff CF24 3AA, U.K.

**Keywords:** Colloidal quantum dots, microlasers, photocleavage, supraparticle, self-assembly, microresonators, whispering gallery modes

## Abstract

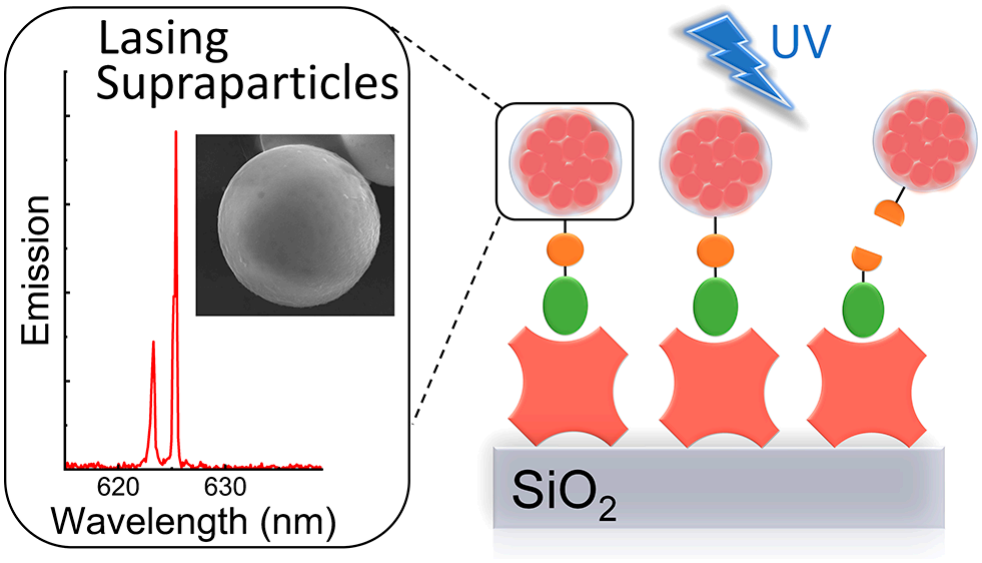

Luminescent supraparticles of colloidal semiconductor nanocrystals can act as microscopic lasers and are hugely attractive for biosensing, imaging, and drug delivery. However, biointerfacing these to increase functionality while retaining their main optical properties remains an unresolved challenge. Here, we propose and demonstrate red-emitting, silica-coated CdS_*x*_Se_1*−x*_/ZnS colloidal quantum dot supraparticles functionalized with a biotinylated photocleavable ligand. The success of each step of the synthesis is confirmed by scanning electron microscopy, energy dispersive X-ray and Fourier transform infrared spectroscopy, ζ-potential, and optical pumping measurements. The capture and release functionality of the supraparticle system is proven by binding to a neutravidin functionalized glass slide and subsequently cleaving off after UV-A irradiation. The biotinylated supraparticles still function as microlasers; e.g., a 9 μm diameter supraparticle has oscillating modes around 625 nm at a threshold of 58 mJ/cm^2^. This work is a first step toward using supraparticle lasers as enhanced labels for bionano applications.

## 1 Introduction

Colloidal semiconductor quantum dots (CQDs) show great promise as effective nanocarriers due to their easily functionalized surface, high photoluminescence quantum yield, and small size resulting in intracellular *in vitro* uptake.^1−3^ Although CQDs are already prominent imaging or luminescent agents, they can be improved by using them as building blocks for mesoscopic supraparticles (SPs). These densely packed microstructures are formed solely of CQDs and are typically a few micrometers in size but can be as small as a few hundreds of nanometers. Although much larger in size compared to single CQDs, micrometer-sized particles still have excellent uptake in tissue.^4−7^ Due to the high refractive index of the CQD material which forms the SPs, the SPs act as optical antennas or resonators and enhance the light absorption and emission compared to individual or dilute CQDs. Furthermore, with the CQDs acting as both the gain material and optical cavity, SPs demonstrate laser oscillation upon optical pumping.^8−11^ In comparison to CQDs, SPs therefore offer enhanced light emission intensity, narrower spectral line widths, and the possibility for refractive index sensing due to the presence of whispering gallery lasing modes, whose properties are affected by changes in the environment at or close to the SP surface.^12^ Consequently, these CQD SPs have great potential for both *in vitro* and *in vivo* biophotonics.

Although laser action from colloidal quantum dot supraparticles is not new, there is a lack of research on the functionalization of these supraparticle lasers. The motivation behind this work is to show how, similar to singular CQDs, CQD SP lasers can be made water soluble and functionalized with a variety of ligands. Therefore, CQD SPs can be used for biophotonic applications just as singular CQDs would be but with the added benefit of enhanced light emission and laser action.

CQD SPs are synthesized via a self-assembly, bottom-up process without the need for expensive equipment using an oil-in-water template technique that utilizes oleic acid functionalized CQDs. Oleic acid is one of the most commonly used capping agents when synthesizing CQDs; therefore, this method can be readily used for a range of commercial or in-house synthesized CQDs. The CQDs are suspended in chloroform and emulsified with an aqueous solution, resulting in spherical SPs with lasing properties. However, due to the oleate molecules, the resultant SPs are insoluble in water, which prevents many of the aforementioned biological applications. To overcome this, we have developed a method to coat the SPs in a silica shell (SP/SiO_2_-NH_2_) which serves three purposes: allows for water solubility; acts as a platform for further functionalization; and potentially reduces the toxicity of the SPs.^8,13^

To demonstrate the possible applications of such a system, we functionalized silica-coated SP lasers with a biotinylated photocleavable ligand (SP/SiO_2_-PC-biotin). Biotin has been used due to its strong affinity to avidin which is commonly used in the development of sensitive assays.^14^ The photocleavable ligand is activated upon UV irradiation (365 nm), which detaches the biotin moieties from the SPs. Using the strong biotin–neutravidin interaction, the microlasers are able to capture neutravidin proteins and then subsequently release them after UV irradiation. The fluorescence intensity is monitored to indicate the binding and release of the functionalized SPs. The difference in fluorescence intensity is so great that it can be simply monitored by using a smartphone camera. We also demonstrate how this system has the capability for lasing, which is retained after full functionalization. Using two different precursors to grow a SiO_2_ shell on the surface of the SP laser results in primary amines on the surface; therefore we can use biomolecules containing *N*-hydroxysuccinimide (NHS) ester terminal groups to covalently couple to the amine groups. This methodology has already been used for the capture and detection of infectious diseases, cancer, and nutrient deficiencies in immunoassays.^15−17^ Combining this modality with a photocleavable group allows for the capture and release of such biomolecules. This novel system could be utilized in biomedical applications, such as point of care diagnostics or even theragnostic agents.^5,18^

## 2 Experimental Section

### 2.1 Materials

Polyvinyl alcohol (PVA), polyvinylpyrrolidone (PVP), tetraethyl orthosilicate (TEOS), photocleavable biotin-PEG3-NHS ester, ammonia (2 M in ethanol), aminopropyltrimethoxysilane (APTMS), and chloroform were purchased from Sigma-Aldrich. CdS_*x*_Se_1–*x*_/ZnS QDs were purchased from CD Bioparticles.

### 2.2 Synthesis of SP/SiO_2_-PC-biotin

#### 2.2.1 Synthesis of SP

The SPs were synthesized by using an oil-in-water emulsion technique. The QDs were suspended in 100 μL of chloroform at a concentration of 20 mg/mL. A 1.25% w/v solution of PVA in 450 μL of Milli-Q water was mixed with the QD solution and vortexed for 5 min. The solution was left to stir at room temperature for 2 h to allow the chloroform to evaporate. The mixture was then centrifuged at 10 000 rpm for 10 min. The supernatant was removed, and the pellet was resuspended in Milli-Q water. The size of the SPs was measured by using SEM images.

#### 2.2.2 Silica Shell Growth (SP/SiO_2_-NH_2_)

The SPs were redispersed in 250 μL of ethanol. PVP was dissolved in water to make a 50 mg/mL solution. In a microcentrifuge tube, 80 μL of SP solution was mixed with 66 μL of PVP solution and sonicated for 20 min. This was repeated a further 2 times. The mixture was centrifuged at 10 000 rpm for 10 min, and the pellet was resuspended in 200 μL of ethanol. The SP-PVP was centrifuged a further 2 times to ensure the removal of any unbound PVP.

TEOS was mixed with ethanol to form a 635 mM solution. The solution of SP-PVP was mixed with 20 μL of the TEOS solution and sonicated for 5 min. After sonication, 800 μL of Milli-Q water and 800 μL of ammonia was added and left to sonicate for 15 min, after which 1.27 μL of a 7.25 mM APTMS solution in ethanol was added. The addition of APTMS was repeated after 30 and 45 min. After an hour had elapsed, the solution was centrifuged at 10 000 rpm for 10 min and the pellet was redispersed in water. This step was repeated 2 more times for purification, resulting in SPs with a silica coating and amine surface groups (SP/SiO_2_-NH_2_).

#### 2.2.3 Functionalization with Biotinylated Photocleavable Ligand (SP/SiO_2_-PC-biotin)

The PC NHS-ester-PEG-biotin ligand was dissolved in anhydrous DMF to make a 50 mM solution. A buffer solution was made by combining 200 mM sodium bicarbonate and 200 mM sodium chloride in Milli-Q water. The resulting SP-SiO_2_-NH_2_ were centrifuged at 13 300 rpm for 10 min, the supernatant was removed and the pellet redispersed in 0.5 mL of the buffer solution to which 88 μL of the PC NHS-ester-PEG-biotin ligand solution was added. The solution was stirred at room temperature for 2 h, centrifuged at 10 000 rpm for 10 min, and redispersed in the buffer solution. Again, this step was repeated a further 2 times for purification.

### 2.3 Fluorescence Sensing Studies

The SPs were resuspended in a solution of 4% BSA; this is to prevent nonspecific binding, and to aid the removal of the SPs during the wash step. Four 5 μL spots of SP solution were drop-cast onto the functionalized glass substrate and left for 3 min before washing off with solutions: 4% polysorbate 20, DI water, and PBS.

### 2.4 Optical Characterization

To test the lasing capabilities of the microspheres, they were pumped with a Nd:YAG, 355 nm, 10 Hz, 5 ns pulsed laser (Minilite, continuum) with a spot size of 2.8 × 10^–5^ ± 0.2 × 10^–5^ cm^2^ measured with a Thorlabs beam profiler. The pump fluence was controlled via a neutral density attenuator with an objective lens (Nikon/10×/0.2NA). A spectrometer (Avantes AvaSpec-2048-4-DT) with a resolution of 0.13 and 0.57 nm was used to acquire the spectra.

SEM images were obtained using an FEI Quanta 250 FEG-SEM and JSM-IT100 scanning electron microscope. EDX spectra were obtained by focusing a beam at the center of two respective spheres. The EDX measurements were performed using FEI Quanta 250 field-emission environmental SEM using an Oxford Instruments 150 mm^2^ silicon drift detector with 30 keV electron beam energy.

The FTIR measurements were obtained with a Nicolet iS5 FTIR Spectrometer, and ζ potential measurements were obtained in water using the dip cell provided for the Malvern Zetasizer Nano ZS.

## 3 Results and Discussion

### 3.1 Synthesis and Characterization of SP/SiO_2_-PC-biotin

**Figure 1:**
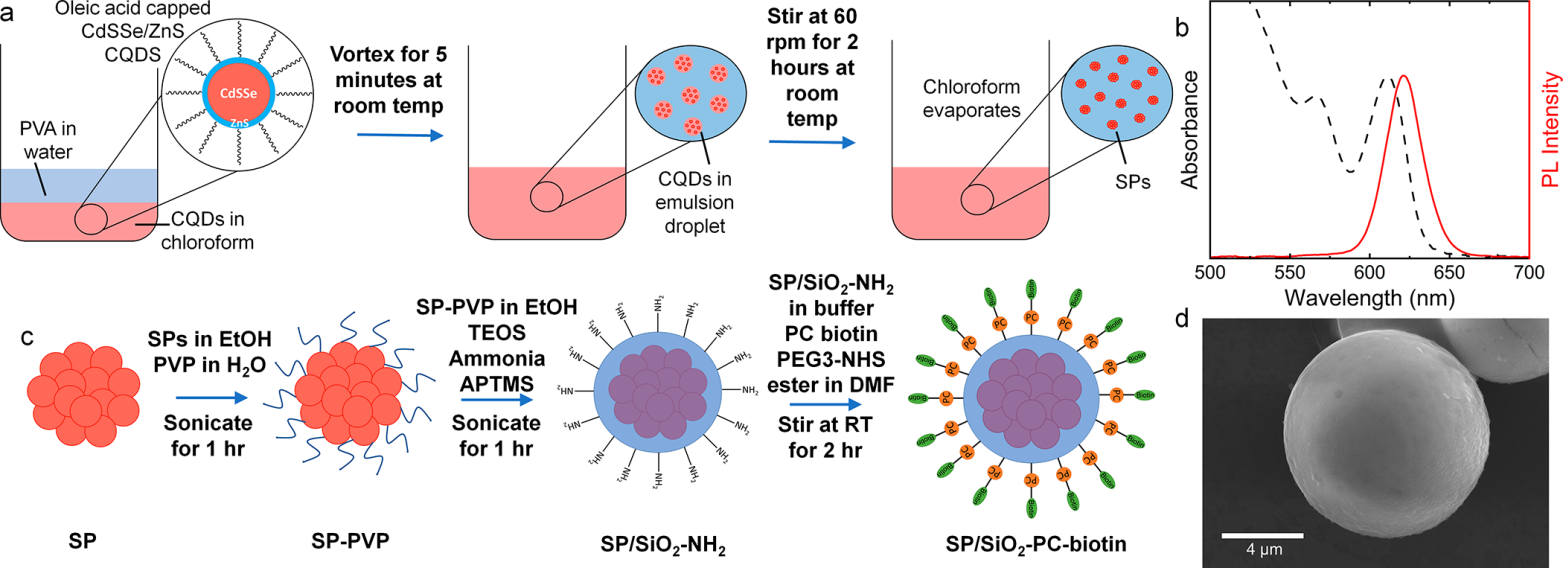
(a) Schematic of oil-in-water emulsion technique used to synthesis SPs from CdSSe/ZnS CQDs. (b) Absorbance and PL emission spectra of CdSSe/ZnS CQDs. (c) Schematic of growth of silica shell using modified Stöber process and surface functionalization with biotinylated ligand. (d) SEM image of SP/SiO_2_-PC-biotin SPs.

The SPs in this work were synthesized using a previously reported oil-in-water emulsion technique demonstrated schematically in Figure 1a.^8,19^ The SPs are composed of oleic acid functionalized red emitting (Figure 1b) CdS_*x*_Se_1–*x*_/ZnS colloidal quantum dots (CD bioparticles) with a diameter of 5.5–6.5 nm as can be seen in the transmission electron microscope image (Figure S1). The oil phase which contains the quantum dots is emulsified with an aqueous solution containing the surfactant polyvinyl alcohol. After a certain amount of time (2–6 h), which is directly related to the size of the emulsion droplet, the oil is evaporated through the water, resulting in densely packed quantum dot SPs of mean diameter 1.2 μm and a standard deviation of 0.5 μm (Figure S2), measured through secondary electron imaging in a field emission scanning electron microscope (FE SEM). Due to the native oleic acid ligands, the SPs are not soluble in water and quickly precipitate. To allow for water solubility, a thin (<5 nm) silica shell is grown on the surface of the spheres using the Stöber method, shown schematically in Figure 1c.^20−22^ This consists of a two-step process: first the native oleic acid ligands are exchanged to polyvinylpyrrolidone (PVP), followed by the deposition of silica in the presence of ammonia. Two silica precursors were used: tetraethylorthosilicate (TEOS) and 3-aminopropyltrimethoxysilane (APTMS). TEOS forms the basis of the silica matrix and APTMS embeds within the silica shell and the surface, resulting in amine groups being present on the surface.^23,24^ The biotinylated photocleavable ligand (PC biotin-PEG3-NHS-ester, Sigma-Aldrich) used contains an NHS ester that allows for the conjugation of the primary amine groups on the SP surface. An SEM image of the fully functionalized SP is shown in Figure 1d. The presence of the silica shell was determined by SEM, energy dispersive X-ray spectroscopy (EDX) and Fourier transform infrared spectroscopy (FTIR). For SEM images of all functionalization steps, see Figures S3 and S4.

**Figure 2:**
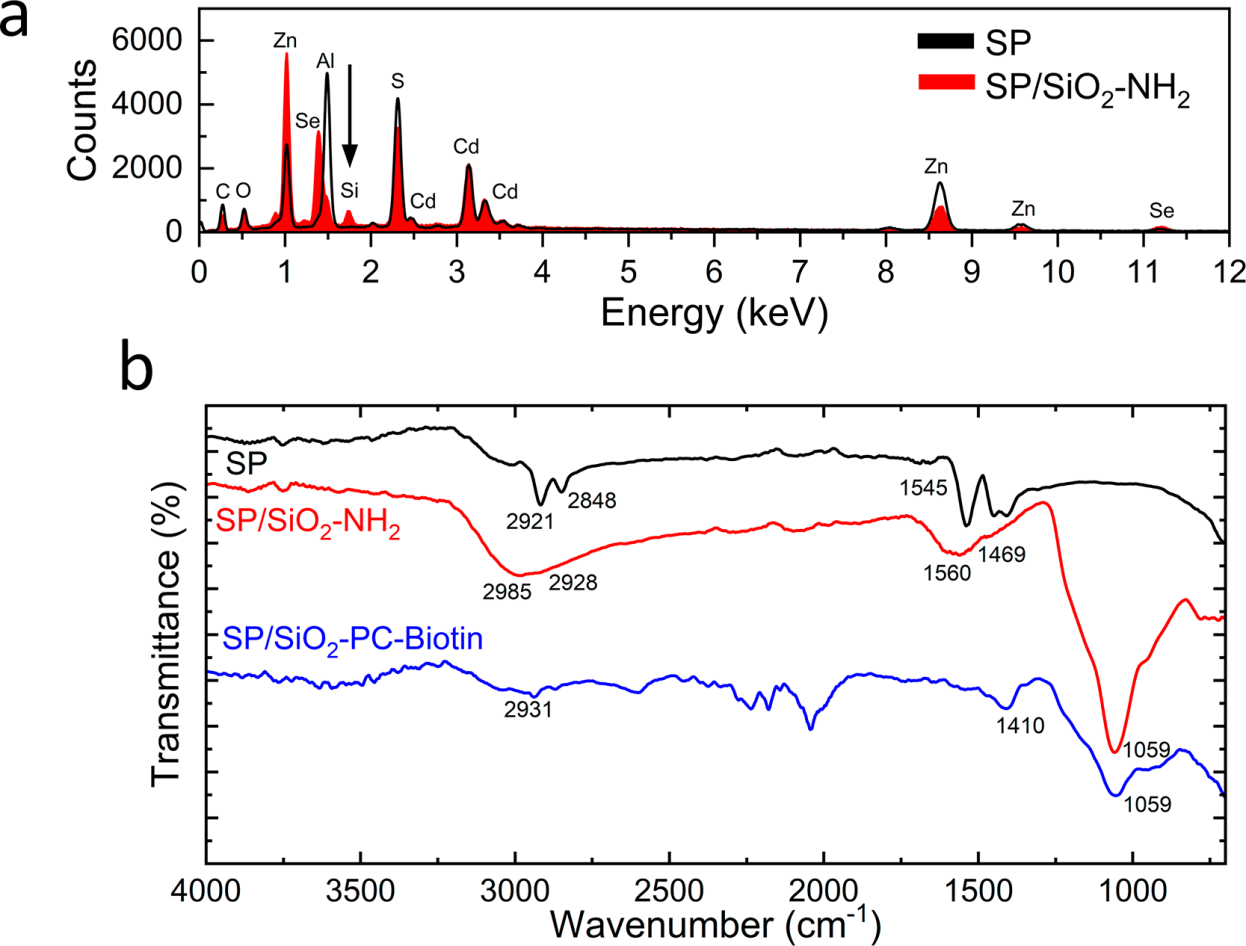
(a) EDX spectra of SP and SP/SiO_2_-NH_2_. (b) FTIR spectra of supraparticle (SP), silica coated (SP/SiO_2_-NH_2_), and biotinylated SP (SP/SiO_2_-PC-biotin).

To ensure the presence of Si was only for the SP, we performed EDX analysis from the SPs drop cast onto an aluminum pin stub. The EDX spectrum (see Figure 2a) shows a peak at 1.7 keV, indicating the presence of Si, which is not present for the uncoated sample. EDX maps (Figure S5) show the distribution of expected elements, such as Cd, S, Zn, and Se, from the CQDs as well as the Si present on the surface of the SP.

FTIR was obtained for all three functionalization steps (Figure 2b). For the SP, the peaks at 2921 and 2848 cm^–1^ correspond to the symmetric and asymmetric CH_2_ stretch of the native oleic acid ligands.^25,26^ After the growth of the silica shell (SP/SiO_2_-NH_2_) a strong peak is present at 1059 cm^–1^, attributed to the asymmetric Si–O–C stretching mode; this band also overlaps with the Si–O–Si mode from the silica shell at 1100 cm^–1^.^27,28^ The broad peak at 1560 cm^–1^ is attributed to the NH_2_ bending vibration, indicating the presence of amine groups on the surface.^29^ Bands at 2985 and 2928 cm^–1^ are from the asymmetric C–H stretching modes from CH_3_ and CH_2_, respectively, and the peak at 1469 cm^–1^ is due to the bending C–H mode from the APTMS.^30−33^ After functionalization with the biotinylated PC ligand (SP/SiO_2_-PC-biotin), the peak at 1059 cm^–1^ remains, indicating that the silica shell is still present. The small peaks at 2931 and 1410 cm^–1^ are from the symmetric stretching of C–H present in the biotin, and the C–H bending mode from the PEG chain in the ligand.^33,34^ In addition to FTIR, the ζ potential of the functionalized SPs was obtained (Table 1). The ζ potential decreases to −44.1 mV after silica shell growth due to the presence of deprotonated silanol groups (SiO^–^) ^35^ and then increases to −27.2 mV after the reaction with the PC-biotin ligand.

**Table 1:** ζ Potential of SP, SP/SiO_2_-NH_2_, and SP/SiO_2_-PC-biotin

sample	SP	SP/SiO_2_-NH_2_	SP/SiO_2_-PC-biotin
ζ potential (mV)	–12.2	–44.1	–27.2
standard deviation (mV)	6.86	9.98	7.83

### 3.2 Fluorescence Sensing

**Figure 3:**
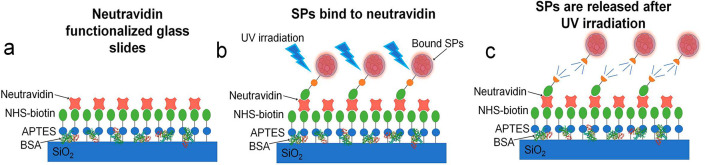
Schematic (not to scale) of the capture and release of biotinylated SPs. (a) The glass slide was functionalized with APTES and NHS-biotin with BSA added to aid nonspecific binding. Neutravidin was then washed over binding to the biotin. (b) The biotinylated SPs are then dropcast, leaving to bind for 3 min before washing. The sample is then irradiated under UV light. (c) The photocleavable groups in the ligand are activated under UV illumination, releasing the SPs which can then be washed away.

To test the added biocapture and release capabilities of the functionalized SPs, a simple sensor was developed to detect the presence of neutravidin (see Figure 3). Glass slides were functionalized with APTES and NHS-biotin.^36^ Neutravidin was washed over the substrate, consequently binding to biotin, with any excess washed off. A control test was run in parallel, where no neutravidin was used. The biotin functionalized SPs were suspended in a 4% solution of bovine serum albumin (BSA) to reduce nonspecific binding. The SP/SiO_2_-PC-biotin was drop cast onto the functionalized slides and left to bind for 3 min before washing to remove any unbound SPs.

**Figure 4:**
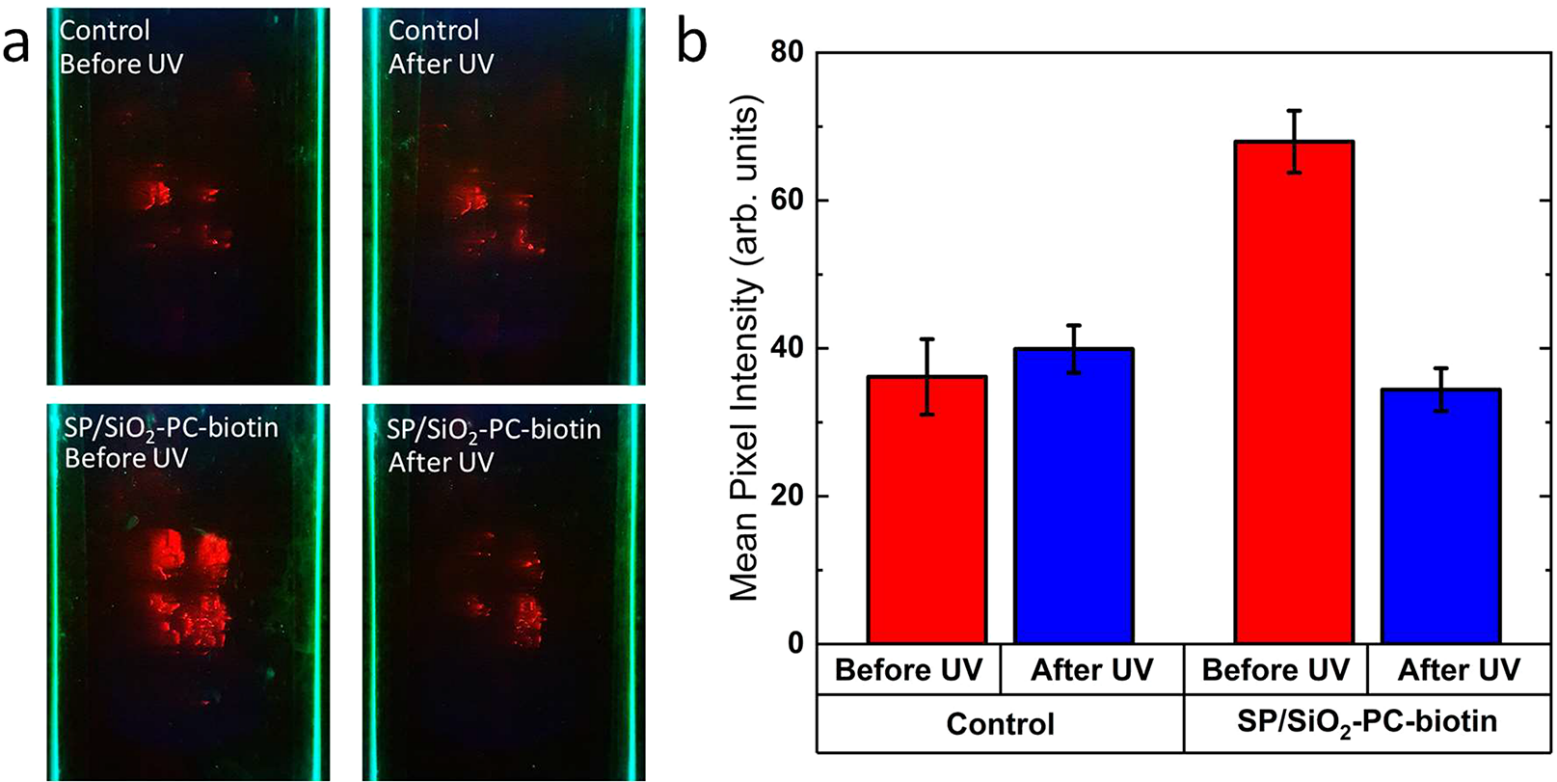
(a) Images of SP/SiO_2_-PC-biotin control (without neutravidin) and SP/SiO_2_-PC-biotin (with neutravidin) before and after UV irradiation. (b) Mean pixel intensity of images from (a). Description of the error bar calculations can be found in Suppporting Information.

The SPs were excited using a 450 nm μLED array which was coupled to a glass slide using the slide as a waveguide, as previously described.^36^ The μLED array was operated with a driving current of 120 mA, which corresponds to an irradiance of approximately 1200 μW/cm^2^ evanescently interacting with the SPs. The fluorescence was imaged using the camera on a smartphone (Samsung Galaxy S9). The camera settings were fixed at ISO 200, 250 ms, 2× zoom, and *f*/1.5 so that the fluorescence intensities could be compared. The sample was then irradiated with a 370 nm UV lamp (UVP B-100) at 5 mW/cm^2^ for 10 min while submerged in a buffer solution (200 mM sodium bicarbonate and 200 mM sodium chloride). The sample was washed and imaged once more. Figure 4a shows the images taken of both the control and functionalized SPs before and after UV irradiation. The reduction in the fluorescence of the SP/SiO_2_-PC-biotin/neutravidin coated sample after UV exposure can be clearly seen from the images. To demonstrate this quantitatively, the fluorescence was measured by calculating the average mean pixel intensity using ImageJ software. The average mean pixel intensity for the control remained the same before and after UV irradiation which suggests that the fluorescence is from SPs that have bound through nonspecific binding. For the sample with specifically bound SP/SiO_2_-PC-biotin, after UV irradiation the fluorescence has decreased 2-fold indicating the ligand was cleaved, releasing the SPs (Figure 4b). The intensity of this SP/SiO_2_-PC-biotin sample after UV is comparable to that of the control measurements.

### 3.3 Microlaser Characterization

**Figure 5:**
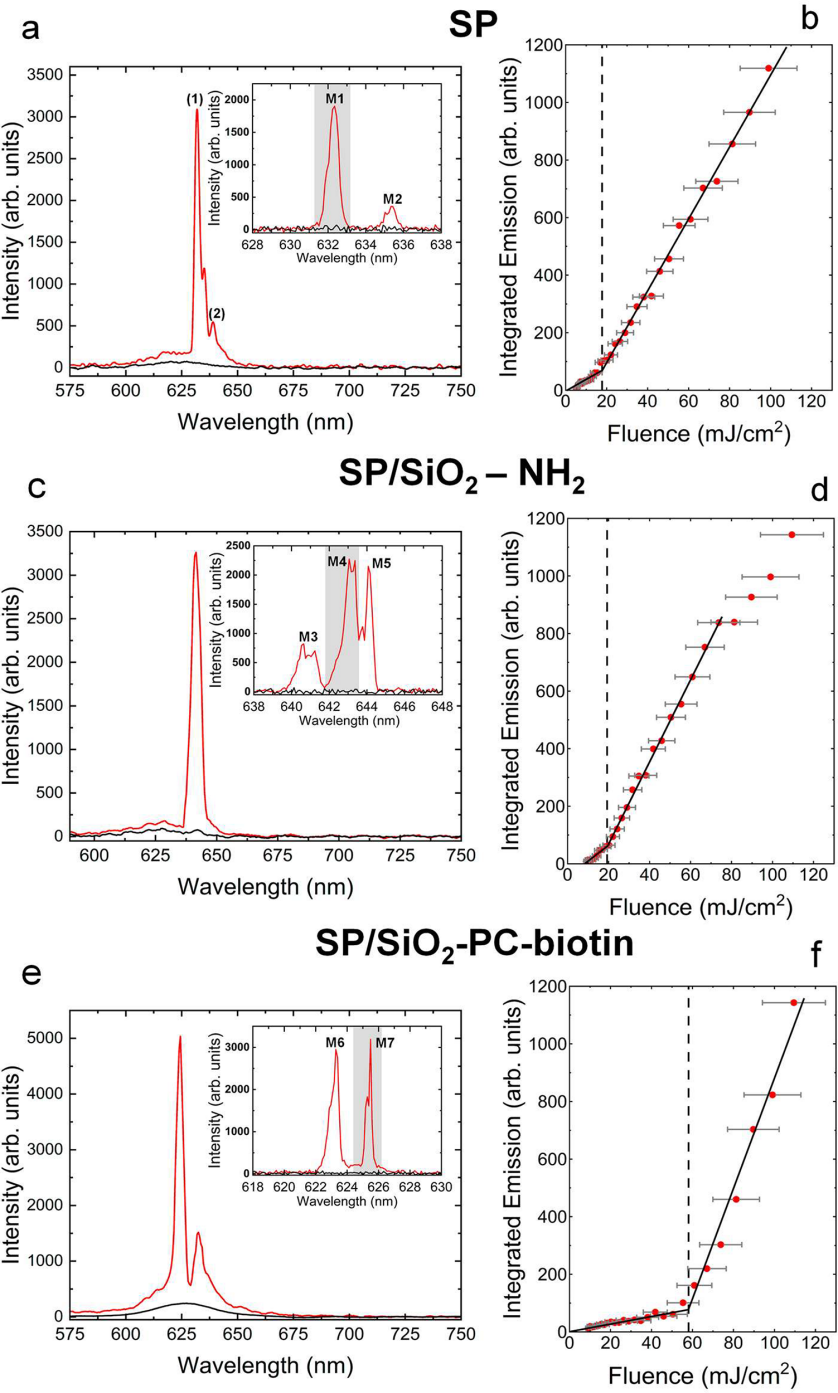
(a, c, e) Emission spectra of a single SP at different pump fluences: (red) 81 mJ/cm^2^ and (black) 11 mJ/cm^2^ pump fluences, with a spectral resolution of 0.57 nm for (a) SP, (c) SP/SiO_2_-NH_2_, and (e) SP/SiO_2_-PC-biotin. Insets for (a), (c), (e): high spectral resolution (0.13 nm) emission spectra revealing substructure. Shaded areas denote areas used for analysis (M1, M4, and M7) for (b), (d), and (f). The diameters of the SPs were 8.9 ± 0.7 μm, 9.3 ± 0.3 μm, 9.6 ± 0.2 μm, respectively. (b), (d) and (f) are the corresponding laser transfer functions of SP, SP/SiO_2_-NH_2_, and SP/SiO_2_-PC-biotin. The thresholds are found to be 17.7 ± 1.7 mJ/cm^2^, 19.3 ± 1.8 mJ/cm^2^, and 58.1 ± 7.6 mJ/cm^2^, respectively. Further information on how the error bars were calculated can be found in Supporting Information.

In Figure 1b, the absorption and photoluminescence spectra of CdSSe/ZnS CQDs in toluene are shown. The emission peak is centered at 625 nm with the first excitonic peak at 610 nm. Using a micro-PL setup, the lasing characteristics of the SPs at different steps of functionalization were investigated by optically pumping individual SPs using a 355 nm Nd:YAG pulsed laser. SPs of similar sizes were pumped for consistency (8.9–9.6 μm). Figure 5a shows PL spectra of a single SP, above and below the lasing threshold. The spectra show evidence of multimode behavior for a pump 3–5 times above threshold as three lasing peaks are observed. This is more evident in Figure 5a inset when measuring at a higher spectral resolution, in which two peaks can be clearly seen at 632.2 nm (M1) and 635.4 nm (M2). The wavelengths of the lasing peaks were also calculated numerically using modal equations. The lasing wavelengths observed experimentally closely match the wavelengths for the transverse electric and transverse magnetic modes found numerically (Figures S6 and S7).

The evolution of the emission intensity as a function of the pump fluence incident on the sample was investigated to determine the threshold characteristics for each of the peaks observed. The threshold for the mode oscillating at 632.2 nm, labeled M1, was found to be 17.7 ± 1.7 mJ/cm^2^ (Figure 5b). Mode M2 began to lase at a higher excitation fluence of 54 ± 7 mJ/cm^2^ at 635.5 nm (Figure S8). The free spectral range (FSR) can be expressed as λ^2^/(2π*an*_eff_), where λ is the resonance center wavelength, *n*_eff_ is the effective refractive index of the mode, estimated to be 1.8, and *a* is the radius of the sphere.^9^ The FSR was obtained from two successive maxima of the same polarization. The peaks denoted (1) and (2) in Figure 5a have an FSR of 6.7 nm. The diameter of the SP thus extracted is 10.2 ± 2.9 μm, which is consistent with the size measured on an optical microscope (8.9 ± 0.7 μm).

Similarly to the SP, a single SP/SiO_2_-NH_2_ showed evidence of multimode lasing (Figure 5c). The mode M4 had the lowest threshold at a pump fluence of 19.3 ± 1.8 mJ/cm^2^ at 643.2 nm (Figure 5d). The thresholds for modes M3 and M5 were found to be 61 ± 8 mJ/cm^2^ and 23 ± 2 mJ/cm^2^ at 640.6 and 644.1 nm respectively (Figure S9).

SP/SiO_2_-PC-biotin had two modes at 623.3 nm (M6) and 625.5 nm (M7), with the lowest threshold at a fluence of 58 ± 8 mJ/cm^2^ for M7 and 89 ± 14 mJ/cm^2^ for M6 (Figure S10). Although the tested samples SP, SP/SiO_2_-NH_2_, and SP/SiO_2_-PC-biotin are similar in size, the wavelength at which the laser oscillation first occurs differs between them: 632.2 nm, 643.2 nm, and 625.5 nm for SP, SP/SiO_2_-NH_2_, and SP/SiO_2_-PC-biotin, respectively. The wavelength of laser oscillation for both SP and SP/SiO_2_-NH_2_ is significantly red-shifted (by 7 and 18 nm, respectively, i.e., more than the FSR) from the peak photoluminescence of 625 nm of the CQDs. The laser wavelength of the SP/SiO_2_-PC-biotin on the other hand is close to the intrinsic CQD PL emission (less than half the FSR difference). The lasing wavelength in these SPs is set by the cavity resonance closest to the net gain peak. The distribution of the lasing wavelength for several spheres of each type of SP was plotted (Figure S11). The mean peak wavelength is 630.7 nm, 629.0 nm, and 623.6 nm for SP, SP/SiO_2_-NH_2_, and SP/SiO_2_-PC-biotin, respectively. Similarly, the laser threshold over a number of SPs was plotted to show the statistical distribution for each sample (Figure S12). The average laser threshold is found to be 15.4 mJ/cm^2^, 26.1 mJ/cm^2^, and 71.9 mJ/cm^2^ for SP, SP/SiO_2_-NH_2_, and SP/SiO_2_-PC-biotin, respectively. For each functionalization step, the average threshold increases while the average peak lasing wavelength decreases. A possible explanation for this could be due to the spectral overlap between the PL emission and absorption of CdSSe/ZnS CQDs (Figure 1b) and the fact that the absorption saturates as the pump excitation increases in CQDs,^37^ this gain material being akin to a 3-level or quasi-3-level laser system. The gain peak wavelength is therefore pump fluence dependent. For the SP/SiO_2_-PC-biotin, the surface is noticeably rougher compared to SP and SP/SiO_2_-NH_2_ (Figures S3). This induces a higher loss for the modes. Consequently, a higher pump fluence is needed to reach lasing threshold, which in turn causes the lasing wavelength to be blue-shifted in comparison to SP and SP/SiO_2_-NH_2_.

Although the laser thresholds appear to be quite high compared to similar systems with thresholds as low as few 10s of μJ/cm^2^,^9,10,38^ it must be noted that these systems were pumped with femtosecond lasers. In comparison, our results are pumped in the nanosecond regime, resulting in higher thresholds. However, for similar systems pumped with nanosecond pulses, the thresholds are in the same range with reports from a 0.23 mJ/cm^2^ to 14 mJ/cm^2^ for unmodified supraparticles.^11,38,39^ The beam spot size of the incident pump in this work is also much greater than the size of the SP; only a fraction of the pump energy therefore interacts and can be absorbed by a SP. The spot size was measured to be 2.8 × 10^–5^ cm^2^ compared to the cross-sectional area of the average studied SP, 6.8 × 10^–7^ cm^2^. If we take into consideration the beam spot size and calculate the percentage of the beam which is incident on the SP, assuming the SP is at the center of the Gaussian beam, then the corrected laser thresholds are 2.9 mJ/cm^2^, 3.2 mJ/cm^2^, and 9.7 mJ/cm^2^ for SP, SP/SiO_2_-NH_2_, and SP/SiO_2_-PC-biotin, respectively. More information about how we calculated this can be found in the Supporting Information. To get an idea of how these fluences would affect biological tissue, we compared a few cases from the literature that use similar lasers, (i.e., pulse duration and wavelength). The reports of biological damage were found at fluences of 100 mJ/cm^2^ for thermal damage of guinea pig skin cells,^40^ and thermal and photochemical damage of the cornea at 1 J/cm^2^.^41^ Therefore, in these cases, laser action would be established for all three types of SP before any damage would occur. However, a higher pump to SP overlap would be preferable to prevent biological damage.

The SP/SiO_2_-PC-biotin is still able to lase after surface immobilization on neutravidin-coated glass. A single sphere of neutravidin bound SP/SiO_2_-PC-biotin was optically pumped under the same conditions as the other SPs and found to retain lasing. For a similar sized sphere (8.2 ± 0.4 μm), the laser threshold was 35 ± 5 mJ/cm^2^ at a wavelength of 632.3 nm (Figure S14). Five individual SPs were optically pumped, and the average threshold was 53.3 mJ/cm^2^ compared to 71.9 mJ/cm^2^ for unbound SP/SiO_2_-PC-biotin. The distribution of the thresholds between samples overlaps (Figure S8); therefore the lower threshold could be due to a number of reasons: a small sample of SPs being measured or the neutravidin functionalized slide acting as a scatterer for the pump energy, resulting in enhanced absorption of the pump energy to the SP, and lower average lasing threshold.

## 4 Conclusion

We synthesized and characterized a supraparticle microlaser platform composed of CdSSe/ZnS quantum dots. The surface of the laser was coated in a silica shell and then further functionalized with a biotinylated photocleavable ligand. After functionalization, the SP retained its lasing properties, with an average lasing threshold of 71.9 mJ/cm^2^. To demonstrate the applications of this system, glass slides were functionalized with neutravidin. The biotinylated photocleavable SPs were bound to the neutravidin, and after UV irradiation, the ligand was cleaved, releasing the SP. This was evidenced by a 2-fold reduction in the fluorescence. These results demonstrate the feasibility of functionalization of such microlasers. Due to the sensitivity to minute changes to its local environment, WGM SPs could be utilized as sensors for biological or defense applications with the ability to add modalities such as drug delivery. CQD SPs are also an attractive alternative to singular CQDs due to their enhanced light emission, lasing properties, and sensing modalities.

## Data Availability

Data set can be found at https://doi.org/10.15129/ff42905f-cfc0-4433-8577-33edec27b721.

## Supporting Information Available

Size distribution and SEM images of SPs for all functionalization steps; EDX elemental maps for SP/SiO_2_-NH_2_; numerical analysis of whispering gallery modes; laser transfer function figures that correspond to laser modes in the high-resolution lasing spectra in Figure 5; distribution of laser threshold and peak emission wavelength for five separate SPs for each functionalization step; lasing spectrum of neutravidin bound SP/SiO2-PC-biotin with corresponding laser transfer function (PDF)
