# Supplementary material for: Biotinylated Photocleavable Semiconductor Colloidal Quantum Dot Supraparticle Microlaser
Source: ACS Appl Nano Mater. 2024 Apr 16;7(8):9159–66. doi: 10.1021/acsanm.4c00668 (PMC11059076; doi:10.1021/acsanm.4c00668)
Supplement: Supplementary file 1 — an4c00668_si_001.pdf [file an4c00668_si_001.pdf]

Supporting Information

Biotinylated Photocleavable Semiconductor Colloidal Quantum Dot Supraparticle Microlaser

Charlotte J. Eling[1], Natalie Bruce[1,2], Naresh-Kumar Gunasekar[3,4], Pedro Urbano Alves[1], Paul R. Edward[3], Robert W. Martin[3], Nicolas Laurand[1]*

[1] Institute of Photonics, Department of Physics, SUPA, University of Strathclyde, Glasgow, G1 1RD, UK

[2] Fraunhofer Centre for Applied Photonics, 99 George Street, Glasgow, G1 1RD, UK

[3] Department of Physics, SUPA, University of Strathclyde, Glasgow, G4 0NG, UK

[4] Institute for Compound Semiconductors, School of Physics and Astronomy, Cardiff University, Cardiff, CF24 3AA, UK

*Corresponding author email: charlotte.eling@strath.ac.uk

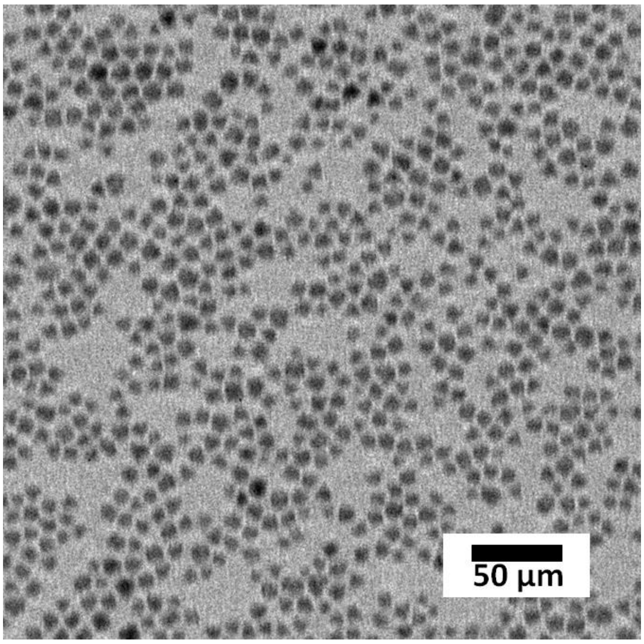

Figure S1: Transmission Electron Microscope image of CdSSe/ZnS QDs.

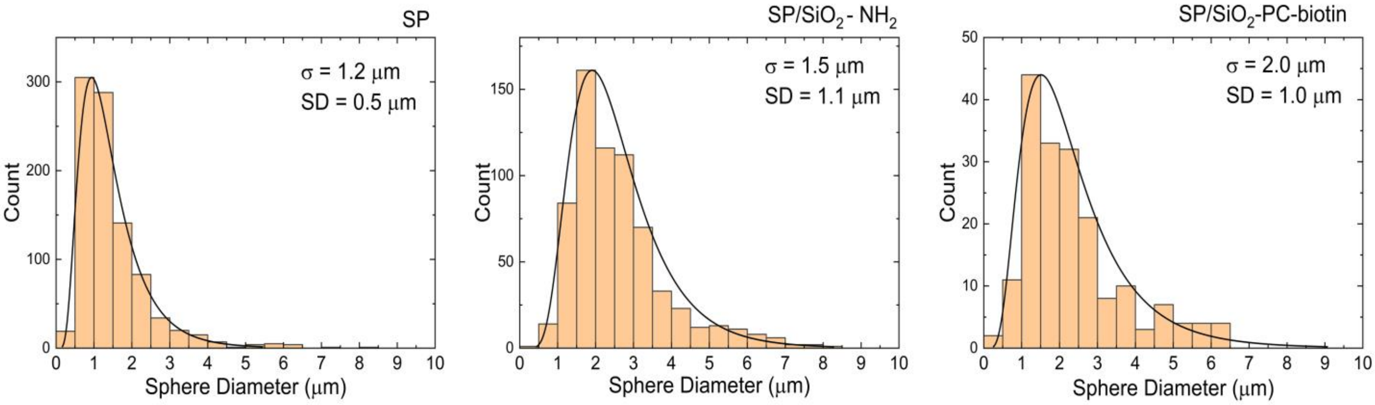

Figure S2: Size distribution of SP, SP/SiO_2-NH_2 and SP/SiO_2-PC-biotin. Size distribution obtained from the secondary electron images obtained in scanning electron microscope. The size distribution of the SPs was fitted with a lognormal curve.

Error bar calculations for Figure 4.

The experiment was repeated 3 times, with 4 x 5 μL of SPs dropcast onto the functionalised substrate. The 4 individual 5 μL spots were highlighted on imageJ software and the mean pixel intensity function was used. The mean value was taken from all 3 repeats of each of the 4 x 5 μL spots. The error bars are the standard deviation of those values.

Error bar calculations for Figure 5, Figures S6, S7, S8 and S10.

The error in fluence considers both the error in measured beam spot size and measured pump energies. The error in fluence was then calculated using the propoagation of error:

$$\frac{\sigma_x}{x} = \sqrt{\left(\frac{\sigma_a}{a}\right)^2 + \left(\frac{\sigma_b}{b}\right)^2}$$

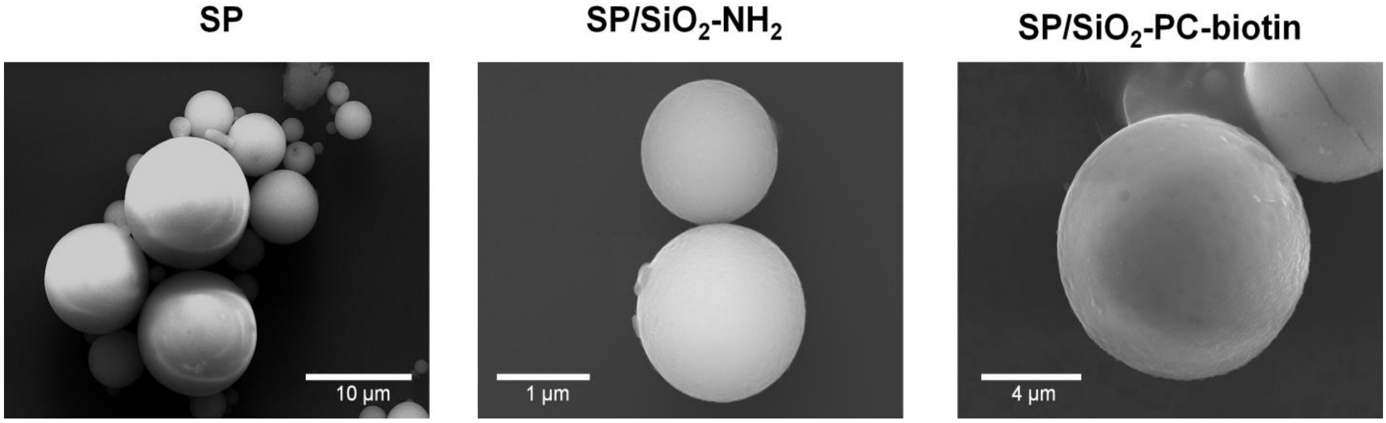

Figure S3: SEM images at different magnifications of SP, SP/SiO_2-NH_2 and SP/SiO_2-PC-biotin.

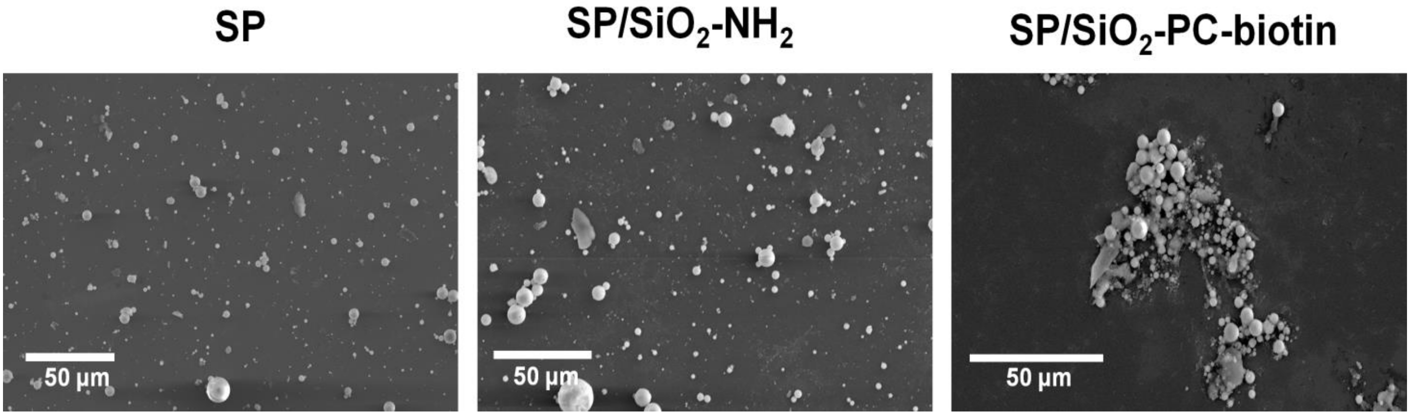

Figure S4: SEM images of SP, SP/SiO_2-NH_2 and SP/SiO_2-PC-biotin.

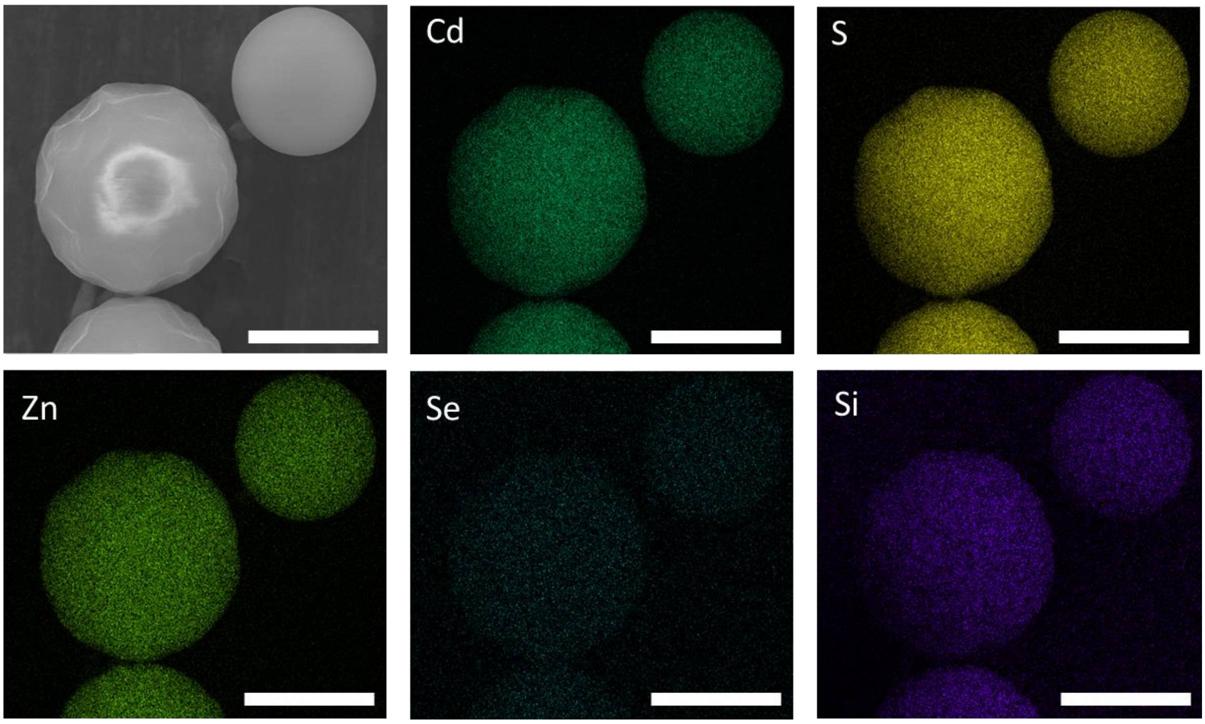

Figure S5: EDX elemental maps of silica coated supraparticles (SP/SiO_2-NH_2). From top left to bottom right; SEM image of supraparticle spheres, map of Cd, S, Zn, Se and Si. Scale bar is 5 µm.

Whispering gallery modes of supraparticles.

The Transverse Electric (TE) and Transverse Magnetic (TM) electric modes of SPs in this work were based on the method written by Stéphane Balac and Patrice Feron (Stéphane Balac, Patrice Feron. "Whispering gallery modes volume computation in optical micro-spheres". [Research Report] FOTON, UMR CNRS 6082. 2014. Hal-01279396v2) and calculated with Wolfram Mathematica™.

The modal equations for TE and TM electric field modes in a microsphere were obtained from the Maxwell equations[1,2]

A sphere with 9.6 µm in diameter (i.e. average size of the SPs reported in this study) and a refractive index of N = 1.7 was used for the calculations[3]. A refractive index of N = 1 was used outside the sphere.

The WGMs are characterized by three parameters referred to as n, l and m, which correspond, respectively, to the number of maxima of the radial, angular and azimuthal field distribution[2]. First, the modal equations for the electric field (TE and TM) were solved for a wavelength close to the observed lasing peaks (λ = 633 nm) in order to determine the l numbers for which the resonances occur (interception of the modal function with 0).

Once the values of l are known, the exact resonance wavelengths of the SP (within the lasing region) can be found. Recurring to the modal equations again, this time using the highest l number within range ($n = 1;\ l = 74$ for TE and $n = 1;\ l = 7$ for TM), we obtain $\lambda = 631\ nm$ for TE and $\lambda = 633\ nm$ for TM (interception of the modal function with 0).

Note that other resonant wavelengths within the lasing region are also possible (e.g. $\{n = 2;\ l = 65\}$, $\{n = 3;\ l = 61\}$, … for TE modes, and $\{n = 2;\ l = 64\}$, $\{n = 3;\ l = 60\}$, … for TM modes), but for the sake of simplicity we will focus on $n = 1$.

Likewise, for illustration purposes the azimuthal field distribution, m, was fixed at $m = 50$ for the TE and TM modes.

Once the parameters above are set, the electromagnetic field can be evaluated and plotted. Figures S1 and S2 show the real components of the Electric field (a.u.) for the modeled sphere.

The effect of the Si shell on the SP surface was neglected due to its thickness (thin layer) and refractive index (N = 1.55 – 1.40), which sits in between CdSSe and air.

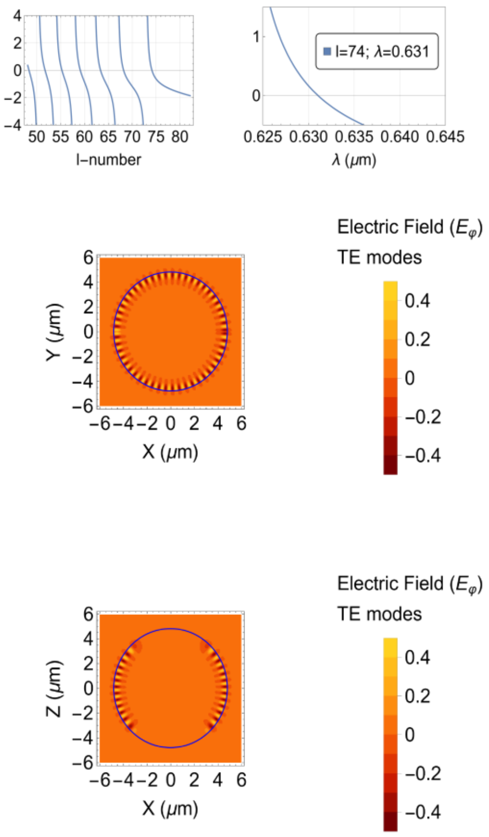

Figure S6: Extracting the l-numbers from the modal equations and finding the respective resonant wavelengths for TE modes (example given for l=74, $\lambda = 631\,nm$). The real component of the Electric field (φ direction, in spherical coordinates) was then calculated at the xy cross section and xz cross section of the sphere (blue circle) for $m = 50$.

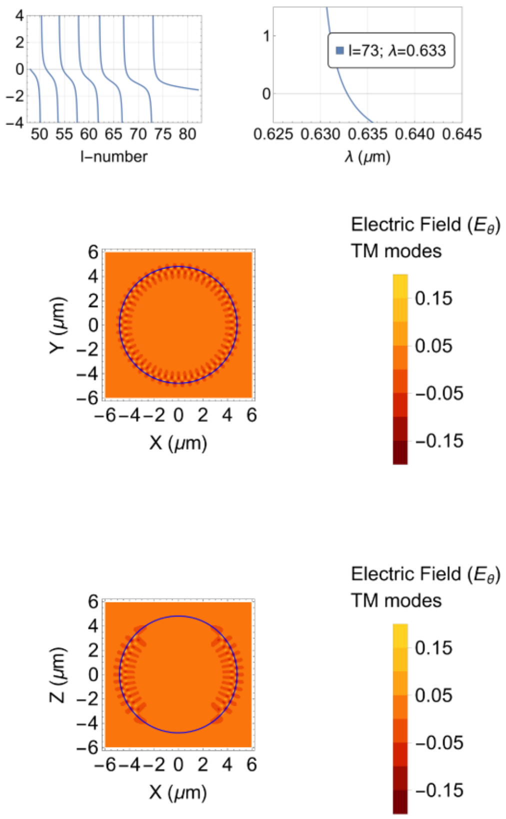

Figure S7: Extracting the l-numbers from the modal equations and finding the respective resonant wavelengths for TM modes (example given for l=73, $\lambda = 633\,nm$). The real component of the Electric field (θ direction, in spherical coordinates) was then calculated at the xy cross section and xz cross section of the sphere (blue circle) for $m = 50$.

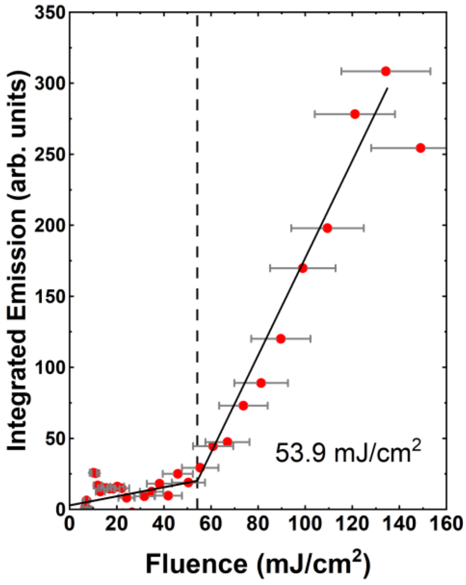

Figure S8: Laser transfer function plot of SP for mode M2. The PL intensity was integrated from 634 nm to 636 nm.

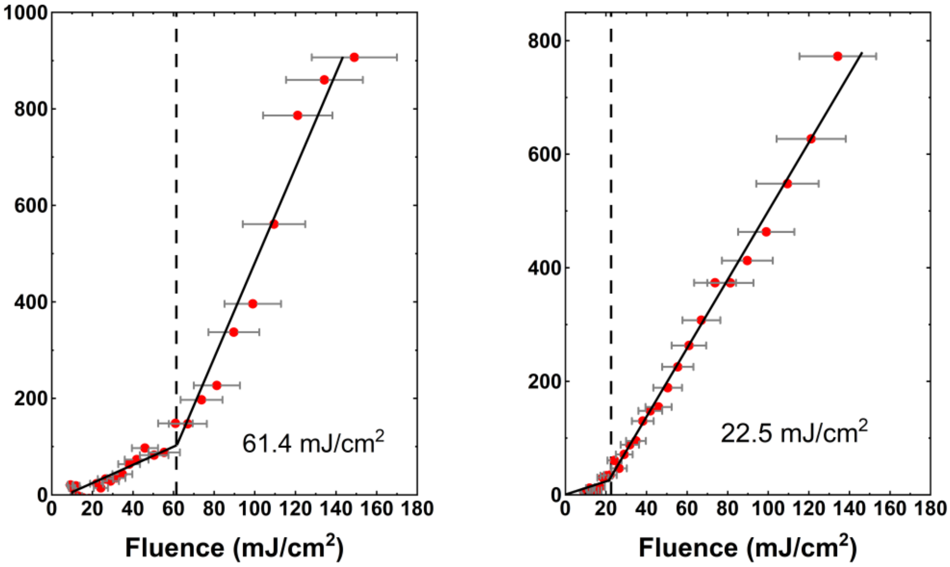

Figure S9: Laser transfer function plots of SP/SiO_2-NH_2. (a) The PL intensity was integrated from 639 nm to 641 nm (M3), (b) 643 nm to 644 nm (M5).

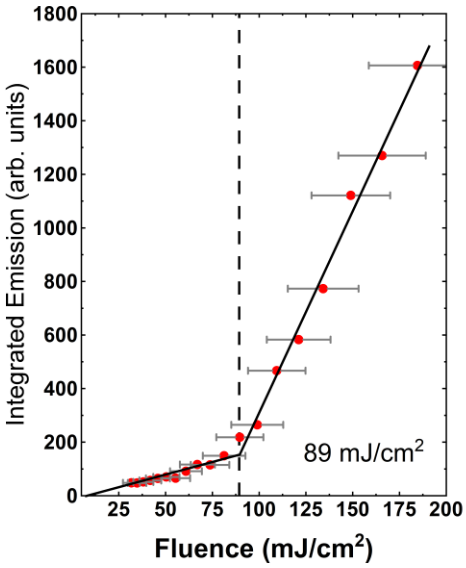

Figure S10: Laser transfer function plot of SP/SiO_2-PC-biotin (M6), the PL intensity was integrated from 622 nm to 624 nm.

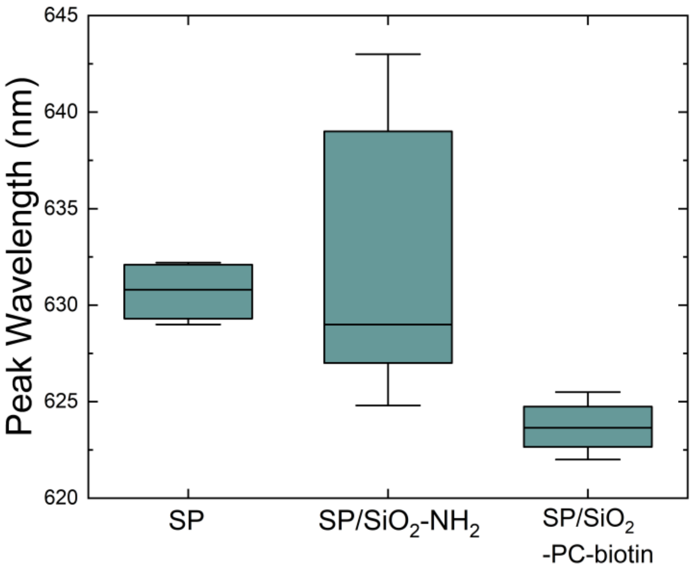

Figure S11: Distribution of peak lasing wavelength for each sample: SP, SP/SiO_2 – NH_2 and SP/SiO_2-PC-biotin. The maxium and minimum wavelengths are shown by the extremities of the plot, the green boxes represent the standard deviation, and the mean is represented by the central line in each green box.

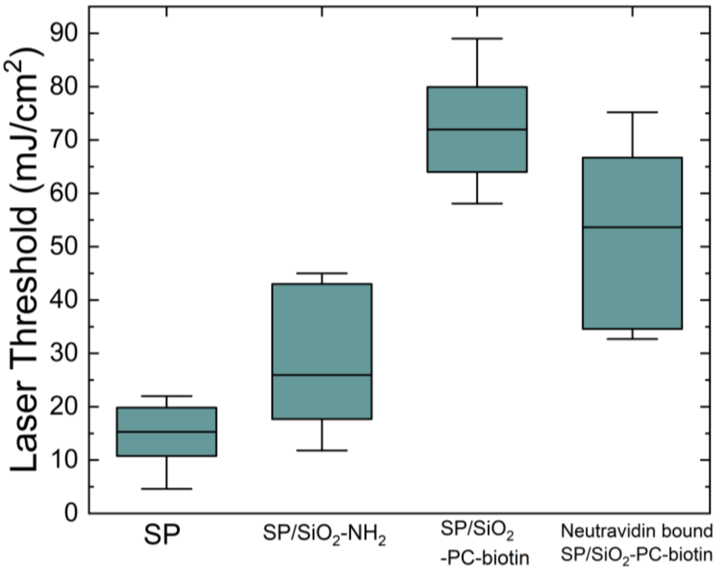

Figure S12: Distribution of laser threshold for five separate SPs for each functionalization step. The maxium and minimum thresholds are shown by the extremities of the plot, the green boxes represent the standard deviation, and the mean is represented by the central line in each green box.

Calculation of lasing thresholds as a proportion of beam spot size.

The beam spot size was measured using a thorlabs CCD beam profiler (BC106N-VIS/M). The beam was elliptical with a beam radius of 39.9 µm and 22.5 µm measured at $1/e^2$ of the intensity. To calculate the beam intensity incident on a SP, the multivariate distribution of the beam was calculated, as can be seen in Figure S10. The double integral was then taken for this function over the size of the SP, assuming the SP lies in the center of the guassian peak. The result show that only 16.7% of the beam intensity would interact with a SP 9.3 µm in diameter.

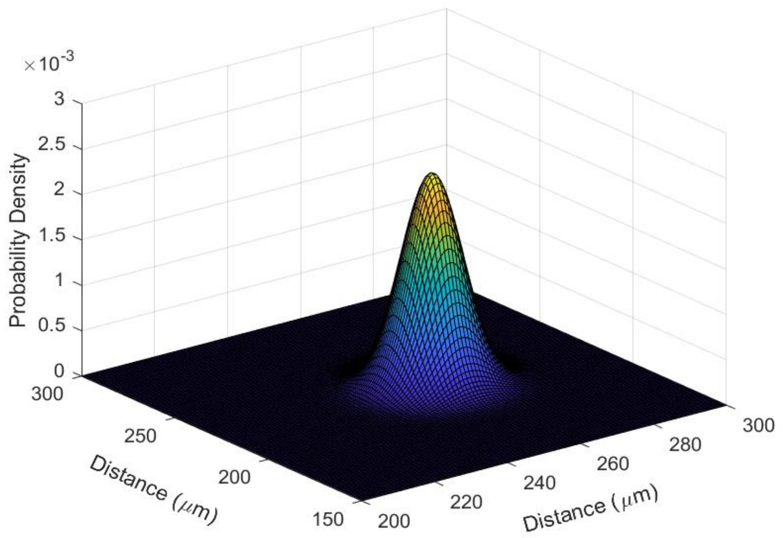

Figure S13: Multivariate normal distribution of incident beam.

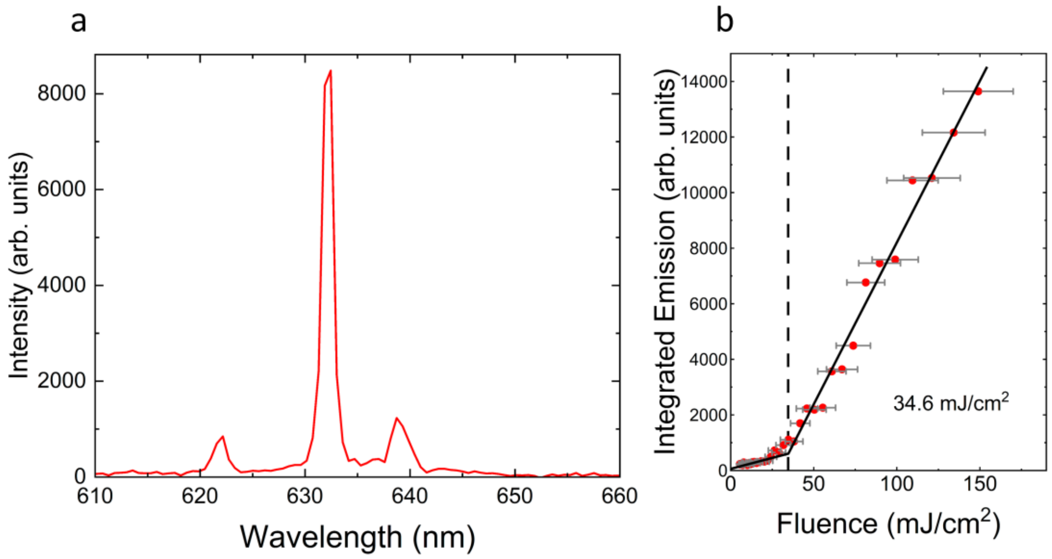

Figure S14: a, Spectra of neutravidin bound SP/SiO_2-PC-biotin. b, Laser transfer function plot of neutravidin bound SP/SiO_2-PC-biotin, the PL intensity was integrated from 629 nm to 635 nm.

References

(1) Alves, P. U.; Guilhabert, B. J. E.; McPhillimy, J. R.; Jevtics, D.; Strain, M. J.; Hejda, M.; Cameron, D.; Edwards, P. R.; Martin, R. W.; Dawson, M. D.; Laurand, N. Waveguide-Integrated Colloidal Nanocrystal Supraparticle Lasers. ACS Applied Optical Materials 2023, 1 (11), 1836–1846. https://doi.org/10.1021/acsaom.3c00312.

(2) Stéphane Balac, Patrice Feron. Whispering gallery modes volume computation in optical microspheres. [Research Report] FOTON, UMR CNRS 6082. 2014. ffhal-01279396v2f

(3) Dement, D. B.; Puri, M.; Ferry, V. E. Determining the Complex Refractive Index of Neat CdSe/CdS Quantum Dot Films. Journal of Physical Chemistry C 2018, 122 (37), 21557–21568. https://doi.org/10.1021/acs.jpcc.8b04522.
